# Supplementary material for: Antibiofilm and Immune-Modulatory Activity of Cannabidiol and Cannabigerol in Oral Environments—In Vitro Study
Source: Antibiotics (Basel). 2024 Apr 9;13(4):342. doi: 10.3390/antibiotics13040342 (PMC11047394; doi:10.3390/antibiotics13040342)
Supplement: Supplementary file 1 [file antibiotics-13-00342-s001.zip › antibiotics-2928494-supplementary.pdf]

Table S1.

Compound		*CBD*					*CBG*					*Peroxide*	*Control*
Concentration		0.25 μM	1 μM	3 μM	10 μM	20 μM	0.25 μM	1 μM	3 μM	10 μM	20 μM	100 μM	
Hours	Parameter												
3	Mean (%)	97.43	96.97	96.37	94.33	94.83	94.8	91.47	88.7	95.63	96.13	0	96.07
	Std. Deviation	0.1528	0.2082	0.05774	0.1528	0.3512	0.2646	0.2517	0.4583	0.1528	0.1528	0	0.2082
	p value	<0.0001	0.0009	>0.9999	<0.0001	<0.0001	<0.0001	<0.0001	<0.0001	0.3494	>0.9999	<0.0001	
6	Mean (%)	95.17	95.1	96.2	97.33	97.5	95.67	95.1	92.9	97.43	97.83	0	94.33
	Std. Deviation	0.5774	0.3606	0.2646	0.4163	0.5	0.2887	0.3606	0.4	0.3215	0.1528	0	0.4163
	p value	0.1173	0.1943	<0.0001	<0.0001	<0.0001	0.0019	0.1943	0.0008	<0.0001	<0.0001	<0.0001	
12	Mean (%)	96.5	95.5	93.73	96.77	96.4	87.5	88.9	84.77	96.5	96.17	0	96.2
	Std. Deviation	0.2646	0.2646	0.2517	0.2517	0.2	0.3	0.2646	0.2517	0.2646	0.5774	0	0.2646
	p value	>0.9999	0.0742	<0.0001	0.2695	>0.9999	<0.0001	<0.0001	<0.0001	>0.9999	>0.9999	<0.0001	
